# An Integrated Machine Learning Framework for Multi-Disease Prediction with Feature Selection

**DOI:** 10.3390/biomimetics11100716

**Published:** 2026-10-08

**Authors:** Jaehyeong Lee, Azam Rakhmatov, Yourim Yoon, Yong-Hyuk Kim

**Affiliations:** 1 Department of IT Convergence Engineering, Gachon University, 1342 Seongnam-daero, Sujeong-gu, Seongnam-si 13120, Gyeonggi-do, Republic of Korea; ljh365365@gachon.ac.kr (J.L.); azamaws1020@gachon.ac.kr (A.R.); 2 Department of Computer Engineering, Gachon University, 1342 Seongnam-daero, Sujeong-gu, Seongnam-si 13120, Gyeonggi-do, Republic of Korea; 3 School of Software, Kwangwoon University, 20 Kwangwoon-ro, Nowon-gu, Seoul 01897, Republic of Korea; yhdfly@kw.ac.kr

**Keywords:** machine learning, health data mining, multi-label classification, feature selection, genetic algorithm, predictive analytics

## Abstract

The coexistence of multiple chronic diseases is common among older adults and presents substantial challenges for disease prevention, management, and healthcare planning. However, most previous studies have focused on predicting individual diseases, although multiple chronic conditions commonly coexist. This study evaluates a multi-output classification model that predicts twelve chronic disease-status labels independently and examines genetic algorithm (GA)- and particle swarm optimization (PSO)-based feature selection using a shared feature subset. Data from the Korean Longitudinal Study of Aging (KLoSA) were analyzed (n=4529). Random forest, decision tree, and XGBoost models were evaluated with five repeated nested holdout splits so that feature selection did not use the outer test set. Across runs, GA and PSO reduced the 131-feature input to 49–77 features, but changes in Composite Macro-F1 were small and model-dependent. High mean label accuracy coexisted with low sensitivity for several rare disease-positive classes. SHAP analysis on held-out data was therefore interpreted descriptively and showed lower ranking stability for selected models. These results indicate a trade-off between feature reduction, predictive performance, and computational cost rather than a general performance advantage or evidence between disease-label interactions.

## 1. Introduction

### 1.1. Background

Advancements in medicine and living standards have significantly enhanced life expectancy, contributing to a global trend of aging populations. South Korea is experiencing this demographic shift at an exceptionally rapid rate, marked by an increase in chronic and degenerative diseases [1]. According to the World Health Organization (WHO), by 2030, individuals aged 60 and over will represent one-sixth of the global population [2]. This demographic shift is contributing significantly to the rise in neurodegenerative diseases, such as dementia. In South Korea, dementia has become a critical social issue, with a notable increase in cases since 2005, a trend that is expected to continue as the population ages [3]. Dementia often co-occurs with chronic conditions like hypertension, diabetes, and cardiovascular diseases, which complicate treatment approaches [4]. This increasing prevalence of comorbidities underscores the necessity for healthcare strategies that address the interactions between dementia and other chronic conditions, such as chronic lung disease, liver disease, psychiatric disorders, arthritis, and rheumatism [4].

Practical dementia care is critical to attaining better health outcomes and reducing the escalating social burden of health care expenditure [5]. Because of the increasing geriatric population, early diagnosis and management of dementia and related ailments have gained immense importance and thus encouraged vibrant research in the field. A multidisciplinary approach, which is mainly based on the early detection of cognitive decline, is fundamental. Multimorbidity is an increasing burden, especially in dementia patients, and places heavy demands on healthcare resources and management systems. Many such patients require the simultaneous management of co-occurring conditions such as hypertension, diabetes, and cardiovascular disease, further necessitating integrated models of care [6]. While some progress has been made regarding such complex health issues, the prevailing approaches often fail to manage multiple chronic conditions simultaneously. Developing healthcare management systems that accommodate multimorbidity is important in addressing the complex medical needs of individuals with coexisting diseases, enhancing overall care beyond isolated disease treatment [7].

However, many existing predictive models primarily focus on single-disease classification, which limits the simultaneous evaluation of multiple chronic conditions within a common experimental framework. Although recent studies have employed Genetic Algorithms (GAs) for feature selection and optimization, their application to multi-output classification remains underexplored [8,9,10]. Building on these prior studies, this study aims to extend feature-selection-based disease prediction to a multi-output setting by evaluating multiple disease-status outcomes in parallel within a unified machine learning framework.

This research assesses health outcomes in the elderly using data collected from the Korean Longitudinal Study of Aging (KLoSA) [11]. This research aims to predict multiple recorded chronic disease-status labels based on shared sociodemographic and health-related features, rather than focusing solely on dementia risk arising from other comorbidities. Because the features and outcomes are measured in the same KLoSA wave, the analysis is cross-sectional and does not forecast future disease onset. A key aim is to compare various algorithms and evaluate feature selection under the same validation procedure. The expected outcomes of this research are therefore interpreted as methodological evidence for multi-disease classification in the studied elderly population rather than as a clinically validated risk model. For this purpose, the GA evaluates a shared feature mask over the 131 features across all twelve independently predicted disease labels. The resulting framework therefore evaluates multiple disease outcomes using a common feature subset without explicitly modeling dependencies among the disease labels. The findings are interpreted as methodological evidence for multi-disease classification in the studied elderly population rather than as validation of a clinical risk model.

The remainder of this paper is organized as follows: In the rest of Section 1, we review related work and remind our experiment and contribution to this study. We introduce our methodology and experimental setup in Section 2 and the results in Section 3. We also discuss our experimental results and contributions to the study across Section 3 and Section 4. Finally, we conclude by summarizing our contribution and limitations in Section 5.

### 1.2. Related Work

As the global population ages, chronic and neurodegenerative diseases, such as dementia, are increasing, exerting considerable pressure on healthcare systems. This trend underscores the critical importance of effective multi-disease management and the development of robust predictive tools. Consequently, there has been a surge in research across various countries focused on forecasting chronic illnesses [12]. This increased research activity demonstrates a global recognition of the need to address these health challenges proactively before they escalate [13,14].

In Japan, studies have employed machine learning techniques to predict hospitalization durations for elderly patients, aiming to enhance healthcare resource allocation and clinical efficiency [15]. In the United States, predictive models such as random forest have been utilized to analyze annual mortality rates and healthcare expenditure patterns among older adults, offering insights to facilitate the early management and prevention of major chronic diseases [16]. Similarly, in Europe, research has focused on understanding the interactions between chronic diseases associated with dementia and developing optimized machine learning-based frameworks for multimorbidity management [17].

These studies emphasize the significance of addressing multimorbidity in aging populations and suggest that early intervention strategies could notably reduce healthcare costs. For instance, research detailed in [18] evaluated risk prediction models constructed on imbalanced datasets, analyzing the impact of chronic diseases on healthcare demand. This study highlighted the considerable burden that conditions like dementia place on healthcare systems, underscoring the urgent need for integrated care models to effectively manage these challenges.

Despite these advancements, existing research [19,20,21] primarily focuses on single-disease prediction models, which limits direct evaluation of multiple chronic disease outcomes within one framework. This research gap motivates predictive frameworks that can evaluate multiple disease variables simultaneously, while the present study treats the individual disease labels as independent prediction tasks.

Furthermore, existing studies often overlook the importance of optimizing feature selection for multi-label classification problems [22]. GA-based feature selection has been used to identify relevant features and reduce model complexity [23], and such data-driven approaches have been discussed in healthcare-oriented predictive modeling [24]. This is particularly relevant to our use of both biological and sociodemographic indicators, an approach aligned with recent studies emphasizing multifactorial data integration [25]. In an imbalanced disease-prediction setting, Rosa et al. reported class-specific sensitivity and specificity together with explainability-based feature investigation [26]. This work provides a related example of why aggregate accuracy should be interpreted together with class-specific measures and why explainability rankings need not coincide with performance-driven feature selection.

To address these gaps, this study aims to enhance the accuracy of prediction models by incorporating multiple indicators from diverse activity domains, addressing existing research gaps. By doing so, it seeks to offer a comprehensive and robust framework for managing chronic diseases among the Korean elderly population. This approach builds on previous research and highlights the importance of holistic care strategies in tackling aging societies’ complex healthcare challenges.

## 2. Experimental Method

### 2.1. Dataset Preparation

This study focuses on developing and evaluating a machine learning model to predict chronic diseases among elderly individuals, using data from the KLoSA [11]. The dataset employed for this study is from the most recent KLoSA survey, released in 2023, derived from the eighth investigation conducted in July to December 2020. It encompasses data collected from about 10,000 middle-aged and elderly individuals born before 1962 who reside in various regions of South Korea, excluding Jeju Island. Although KLoSA is a longitudinal panel study, each wave is collected as an independent cross-sectional dataset, with partial replacement of respondents due to attrition and sample replenishment. Therefore, earlier waves (1st through 7th) were not merged for dataset in this study. A detailed summary of the survey items is presented in Table 1.

In this study, the raw data from the KLoSA underwent significant preprocessing to ensure analytical rigor before being used for analysis. Unique identifiers such as IDs, interview status, and dates were removed from the dataset, as they were irrelevant to the model’s predictions. Response prompts and questions that varied based on employment status were also excluded to avoid unfairly influencing the predictive outcomes. The focus was on health-related questions, specifically retaining only current health status information while excluding time-series data like the age of disease onset, which was outside the study’s scope.

Cognitive ability indicators were defined to include factors such as dementia (memory and thinking), intelligence (IQ), ADHD (attention deficit and hyperactivity), and language skills (speaking, listening, reading, and writing), which are essential for daily activities, learning, and social interactions. To reduce redundancy, representative variables were selected when similar items were present; for example, only BMI was retained from variables such as BMI, weight, and height. Subjective job expectancy, which varies across age groups, was consolidated into a single composite variable. Additionally, multi-label data was converted into an appropriate format to ensure that all relevant health indicators were properly formatted for inclusion in the model. These steps were critical in preparing the dataset for a robust and reliable machine-learning analysis to predict chronic diseases among the elderly.

Given the multi-label nature of the chronic disease items in this study, they were encoded using multi-hot encoding [27]. In the source KLoSA data, response code 1 indicates disease presence and code 5 indicates disease absence; these values were recoded to modeling values 1 and 0, respectively. Eleven records with dementia response code 3 were excluded because that response could not be assigned unambiguously to either binary class. This encoding method enabled the machine learning models to handle multi-output prediction problems effectively. After preprocessing and target validation, the dataset included 131 feature variables and 12 target labels (143 analytical columns in total) across 4529 respondents. Details on feature-wise missingness and preprocessing outcomes are provided in Appendix A, and Supplementary Material S1 lists features and targets separately.

For the multi-label classification task, we considered a wide range of target conditions, including hypertension, diabetes or hyperglycemia, cancer and malignancy (excluding minor skin cancers), chronic lung disease, liver disease, heart disease, cerebrovascular disease, psychiatric disorders, arthritis or rheumatism, digestive system diseases noted since the last baseline survey, disc disease noted since the last baseline survey, and dementia. Table 2 reports disease-positive and disease-negative counts and the corresponding disease-positive prevalence. Because features and disease labels were measured in the same survey wave, this study evaluates contemporaneous disease-status classification rather than future disease-onset prediction; possible same-wave proxy information is considered as a limitation.

### 2.2. Configuration of Analytical Methods and Models

We conducted experiments following two distinct stages. First, the analysis in this study involved the data-cleaning process described in Section 2.1. Variables with more than 20% missingness and records remaining incomplete after this rule were removed as fixed study-level eligibility criteria before model evaluation. This produced 4540 complete records; a total of 11 additional records with dementia response code 3 were then excluded, leaving 4529 respondents. Because these missingness-based eligibility rules were applied before the repeated model-evaluation splits, the full-cohort missingness pattern could affect variable and record inclusion, which is acknowledged as a limitation. For details of the feature set used after data preprocessing is complete, please refer to Supplementary Material S1.

**Figure 1:**
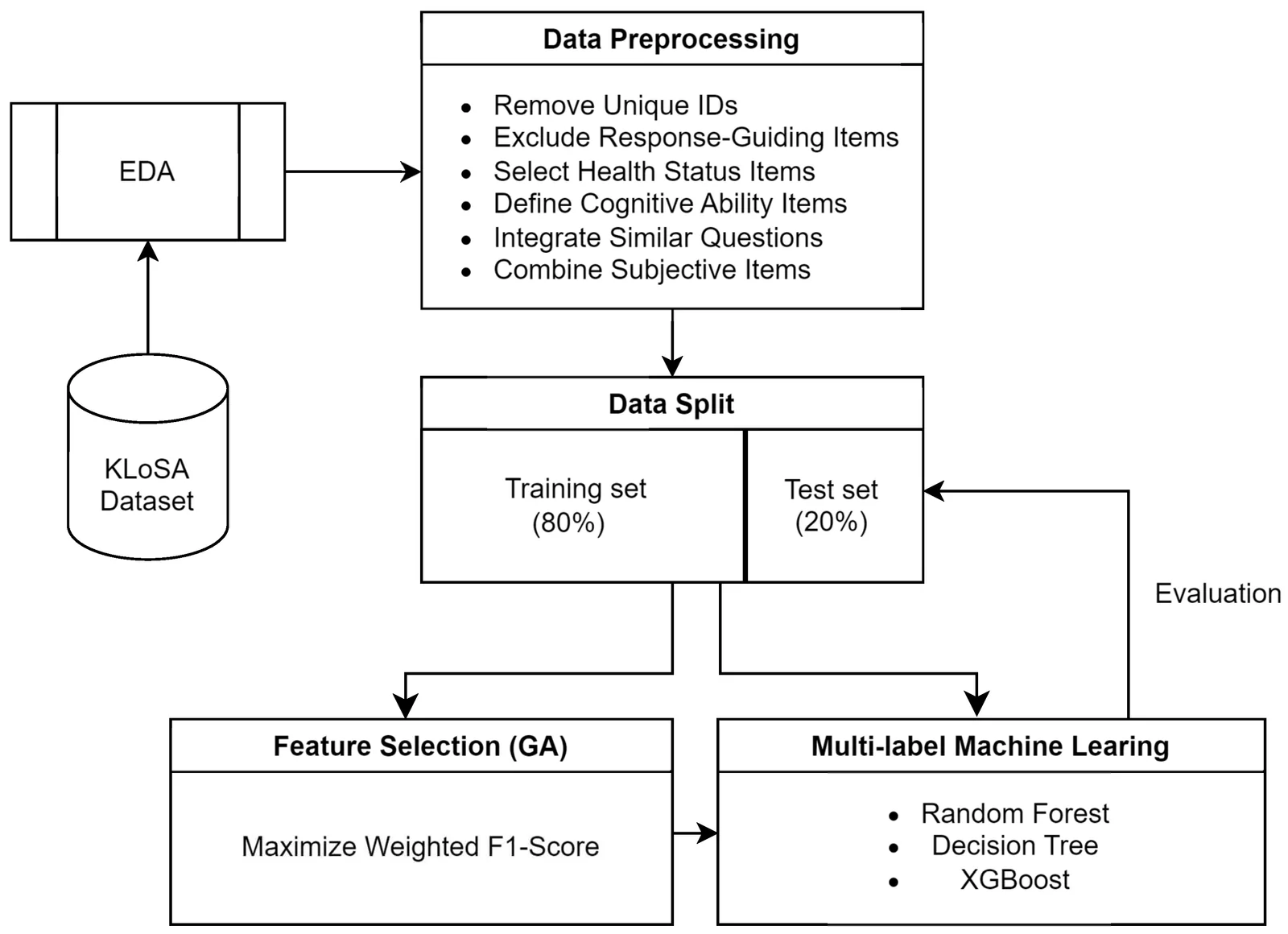
Summary of dataset preparation and preprocessing process.

In the second stage, model evaluation used five repeated nested holdout splits with outer five seeds. For each seed, 3623 records formed the outer development set and 906 formed the held-out outer test set; the development set was further divided into 2898 inner-training and 725 inner-validation records. GA and PSO fitness evaluation, GA mutation-rate selection, and feature-subset selection were confined to the inner data. After the subset was fixed, the estimator was refitted on the complete outer development set and evaluated on the outer test set. The selected features were evaluated using random forest [28], decision tree [29], and XGBoost models with the multi-output classifier [30] for multi-label classification problems. No outer test score was used for feature or mutation-rate selection. The overall process is summarized in Figure 1.

### 2.3. Applying the Multi-Output Classifier

We applied the multi-label classification problem using the multi-output classifier, implementing both random forest and decision tree models based on this framework. Multi-label classification involves predicting several target labels simultaneously for a single sample. Each label is explicitly treated as an independent binary classification task rather than being modeled jointly. This approach does not account for inter-label dependencies but provides a transparent baseline framework for independent multi-label prediction, which is appropriate for the scope of this study.

The multi-output classifier framework supports this by training a separate classifier for each target label, adopting a modeling strategy that assumes and maintains independence among target variables. Each label is treated as an individual binary classification task, with an independent classifier dedicated to it. All twelve disease estimators use the same feature matrix, but no disease label is used as an input for another label and no shared phase is trained. This independence assumption allows us to isolate per-disease performance without inferring interactions among diseases. Thus, this methodology is particularly apt for scenarios like multi-disease prediction, where conditions such as dementia, hypertension, and diabetes may need to be predicted simultaneously. These conditions are considered independently, thereby enabling a more precise evaluation of each disease’s prediction performance [31].

In this study, we defined each target label as a distinct binary classification task and trained independent classifiers using the random forest and decision tree through the multi-output classifier. This approach allowed us to generate independent predictions for each disease, effectively addressing the study’s inherent multi-label nature, where multiple diseases may coexist. We employed a multi-output classifier that trains a separate binary classification estimator for each disease label, while clearly maintaining label independence rather than modeling correlations.

We calculated class-specific and aggregate metrics for each disease during the evaluation phase and compared random forest, decision tree, and XGBoost under the same repeated holdout splits. Because several targets are highly imbalanced, accuracy is interpreted together with disease-positive precision, recall, and F1-score. Composite Macro-F1 and PR-AUC are retained as aggregate summaries in Section 2.6. This methodology preserves the target-wise comparison while avoiding reliance on accuracy alone.

### 2.4. Machine Learning Algorithms

In this study, we conducted the analysis using XGBoost, random forest, and decision tree models. The decision tree is retained as a simple single-tree reference, whereas random forest and XGBoost are ensemble learners. Parameter values used in this study are summarized in Table 3. In general, deep learning models are known to perform well, but for tabular data like the survey data we use, tree-based models, or boosted models, are known to be better than deep learning models and perform better. These models also have a time advantage over deep learning [32]. Therefore, we decided to use tree-based models for training.

#### 2.4.1. Random Forest Classifier

The Random Forest Classifier is an ensemble learning method that utilizes multiple decision trees to form a robust model. Each tree within the forest is trained independently through bootstrap sampling and selecting a random subset of features, contributing to the diversity among the trees. The final prediction is made based on majority voting from all the trees. This method not only reduces overfitting but also ensures that the predictive models are stable and reliable, instilling confidence in the audience about the robustness of model [33].

#### 2.4.2. Decision Tree Classifier

The Decision Tree Classifier is a classification method that uses the decision tree algorithm. In our study, we implemented this classifier using the Scikit-Learn library, a popular machine learning toolkit. The classifier works by partitioning the data into branches based on decision rules derived from the data’s features. Each decision point, or node, splits the data based on a condition, leading to the leaf nodes where the final class labels are assigned. This process creates an intuitive and easy-to-interpret model, making it particularly useful for tasks where understanding the decision logic is important [34].

#### 2.4.3. XGboost Tree Classifier

The XGboost Tree Classifier is a powerful classification method based on the Gradient Boosting framework, which builds an ensemble of decision trees to make predictions. Each tree corrects the errors of the previous one, and the final prediction is made by combining the outputs of all trees. XGBoost incorporates regularization to prevent overfitting and improve generalization. It is efficient, scalable, and handles complex datasets well, making it highly effective for classification tasks [35].

### 2.5. GA-Based Feature Selection

A GA is an optimization technique inspired by the principles of natural selection and are particularly useful in addressing complex computational problems. The process begins by randomly generating an initial population of candidate solutions, and each is evaluated based on a fitness function that measures its effectiveness. The candidates with the highest fitness scores are then selected for reproduction through genetic operators such as crossover and mutation. This iterative selection, crossover, and mutation process continues across generations, aiming to converge toward an optimal or near-optimal solution [36,37].

Population size represents the number of individuals maintained in each generation of a genetic algorithm, where a larger value allows for broader solution space exploration but increases computational cost. Typically, values between 50 and 500 are used depending on the complexity of the problem. Generations refer to the total number of iterations the algorithm will run, where more generations increase the likelihood of finding better solutions but also extend computation time. Mutation rate represents the probability that genes will be randomly altered in a new generation, where a low mutation rate (0.001–0.05) promotes stable convergence but risks getting stuck in local optima, while a high mutation rate increases diversity but makes it difficult to maintain good solutions [36,38].

In feature selection, especially for multi-label classification problems, GAs can evaluate candidate feature subsets against several prediction targets [38,39]. In this study, each binary chromosome represents inclusion or exclusion of the same 131 features for all twelve independently fitted disease estimators. The shared mask allows one subset to be evaluated across the twelve outputs, but it does not model interactions among the disease labels [38].

GAs, when performing feature selection, search different combinations of features and have been used as part of data-driven optimization procedures [40]. Furthermore, GAs test various combinations of features and identify combinations according to the specified fitness function. Because GAs is a type of metaheuristic, they can be applied as feature selection for models that predict several diseases with filter, wrapper, and embedded methods [41]. In the present experiment, the fitness function evaluates predictive performance rather than explicit disease-label interactions [41].

Thus, this study implements GA-based feature selection to examine whether a shared feature subset can reduce the input dimension while maintaining predictive performance. The parameters used in this study are presented in Table 4. Specifically, the GA was implemented with a binary chromosome encoding representing the inclusion or exclusion of each of the 131 features. The population size and maximum number of generations were both set to 100, with mutation rates of 0.005, 0.01, and 0.05 evaluated separately for each learner and outer seed; the rate and feature mask were selected using only inner-validation data. The fitness function was defined as the weighted positive-class F1 score obtained from the wrapper model trained on the selected feature subset. The objective does not include an explicit penalty on subset size and is not treated as an imbalance-corrected metric. Elitism was then applied to preserve the top-performing individuals in each generation.

In contrast, for the comparison with GA, we also implemented Particle Swarm Optimization (PSO) [42], which is widely utilized metaheuristic algorithm such as GA. PSO used the same 131-feature mask representation and inner-validation objective. The parameters used in this algorithm are summarized in Table 5. Both algorithms used the fixed maximum budgets shown in Table 4 and Table 5; adaptive early stopping was not evaluated.

### 2.6. Evaluating Model Performance

To evaluate model performance, we use accuracy, disease-positive precision, recall, and F1-score at the target level, together with Composite Macro-F1 and Precision–Recall Area Under the Curve (PR-AUC) as aggregate summaries. Accuracy provides a conventional measure of overall label-wise correctness, but it is interpreted together with class-specific metrics because several disease labels are highly imbalanced [43]. Disease presence is treated as the positive class throughout the evaluation.

#### 2.6.1. Macro F1-Score

Macro F1 gives equal importance to the positive and negative classes within each disease target [44,45]. For each disease, F1 is calculated separately for the positive and negative classes, and the two values are averaged. Composite Macro-F1 is then the arithmetic mean of these twelve target-level macro F1 scores. These metrics are calculated by (1) and (2):

(1) $$F_{1,j}^{\left(+\right)}=\frac{2TP_{j}}{2TP_{j}+FP_{j}+FN_{j}},\;F_{1,j}^{\left(-\right)}=\frac{2TN_{j}}{2TN_{j}+FP_{j}+FN_{j}},$$

(2) $$\mathrm{Composite}\;\mathrm{Macro}\text{-}F1=\frac{1}{12}\sum_{j=1}^{12}\frac{F_{1,j}^{\left(+\right)}+F_{1,j}^{\left(-\right)}}{2}.$$

This two-stage averaging gives equal weight to the positive and negative classes within each disease and equal weight to all twelve disease targets.

#### 2.6.2. Weighted F1-Score

A support-weighted F1 assigns larger weights to more frequently represented outcomes [46]. In this study, it is used only as the inner-validation objective for GA and PSO feature selection, which is calculated by (3):

(3) $$F1_{\mathrm{weighted}}=\sum_{j=1}^{12}\frac{n_{j}^{\left(+\right)}}{\sum_{k=1}^{12}n_{k}^{\left(+\right)}}F_{1,j}^{\left(+\right)},\;n_{j}^{\left(+\right)}=TP_{j}+FN_{j}.$$

Because this objective gives greater weight to more common positive labels, it is not treated as a headline imbalance-aware performance measure.

#### 2.6.3. Precision–Recall Area Under the Curve (PR-AUC)

PR-AUC is calculated from the saved probability scores for each disease and then averaged across the twelve targets. It provides a threshold-independent summary of precision–recall behavior under class imbalance. All reported metrics are first calculated for each outer seed and then summarized as mean and sample standard deviation across seeds 42–46. Because the repeated outer splits overlap, these values are used descriptively and no inferential significance test or confidence interval is reported.

## 3. Result

### 3.1. Machine Learning Performance Evaluation

**Figure 2:**
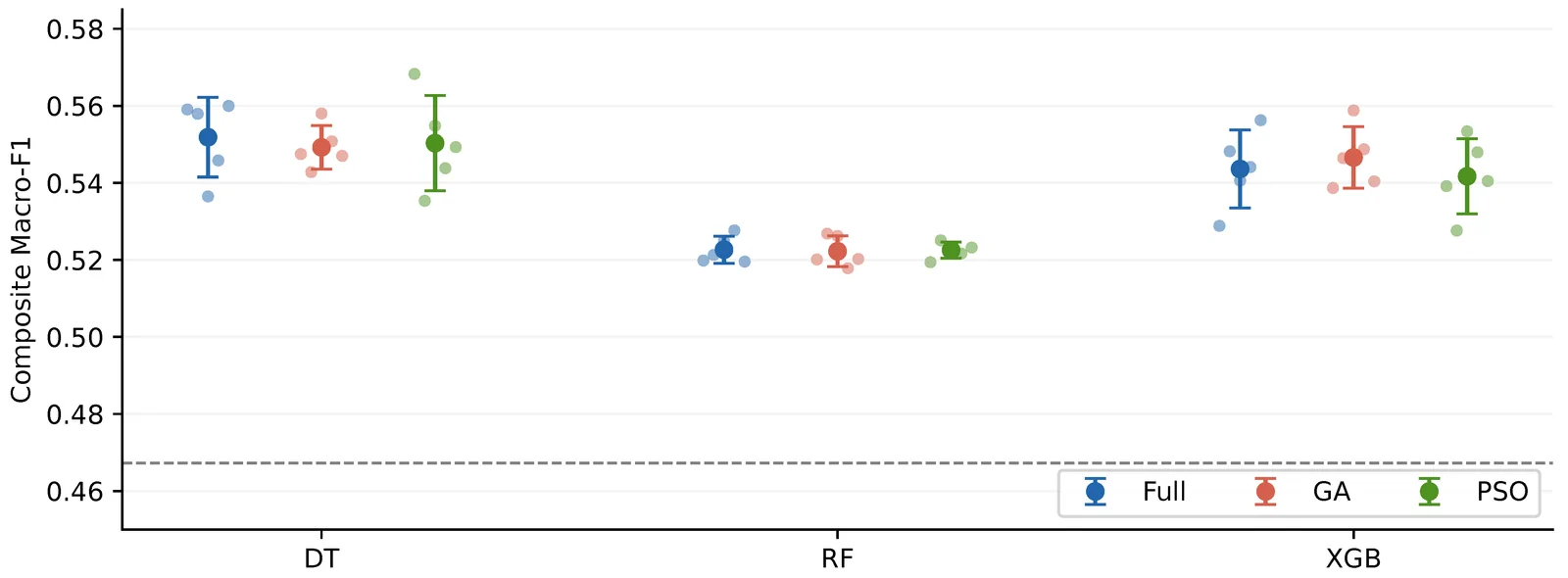
Composite Macro-F1 for the full, GA-selected, and PSO-selected models across the five outer seeds.

Table 6 and Figure 2 reports accuracy, disease-positive precision, recall, and F1-score for each target, followed by Average Accuracy, Composite Macro-F1, and PR-AUC. The all-negative reference baseline achieved an average accuracy of 0.888 while detecting no disease-positive cases, and the prevalence-matched reference baseline achieved an average accuracy of 0.837. These results show why accuracy alone is insufficient for the highly imbalanced targets.

Composite Macro-F1 for the full Decision Tree, Random Forest, and XGBoost models was 0.552±0.010, 0.523±0.004, and 0.544±0.010, respectively, with corresponding Macro PR-AUC values of 0.140±0.008, 0.249±0.014, and 0.260±0.019. After GA and PSO selection, both summaries changed only slightly and in a model-dependent manner, and neither feature-selection method showed a uniform performance advantage across learners.

### 3.2. Analysis for GA-Based Feature Selection

The GA and PSO results are interpreted as a feature-reduction trade-off rather than evidence that a minimal or uniquely necessary subset was identified. Across the five outer seeds, GA retained 49–74 features and PSO retained 50–77 of the 131 features. Mean GA subset sizes were 69.4, 55.4, and 59.8 for Decision Tree, Random Forest, and XGBoost, respectively. The corresponding PSO means were 65.8, 60.0, and 68.2. Table 7 also reports the recorded number of fitness evaluations and search time. For GA, the reported search cost sums the three mutation-rate searches, whereas PSO reflects its single search.

**Figure 3:**
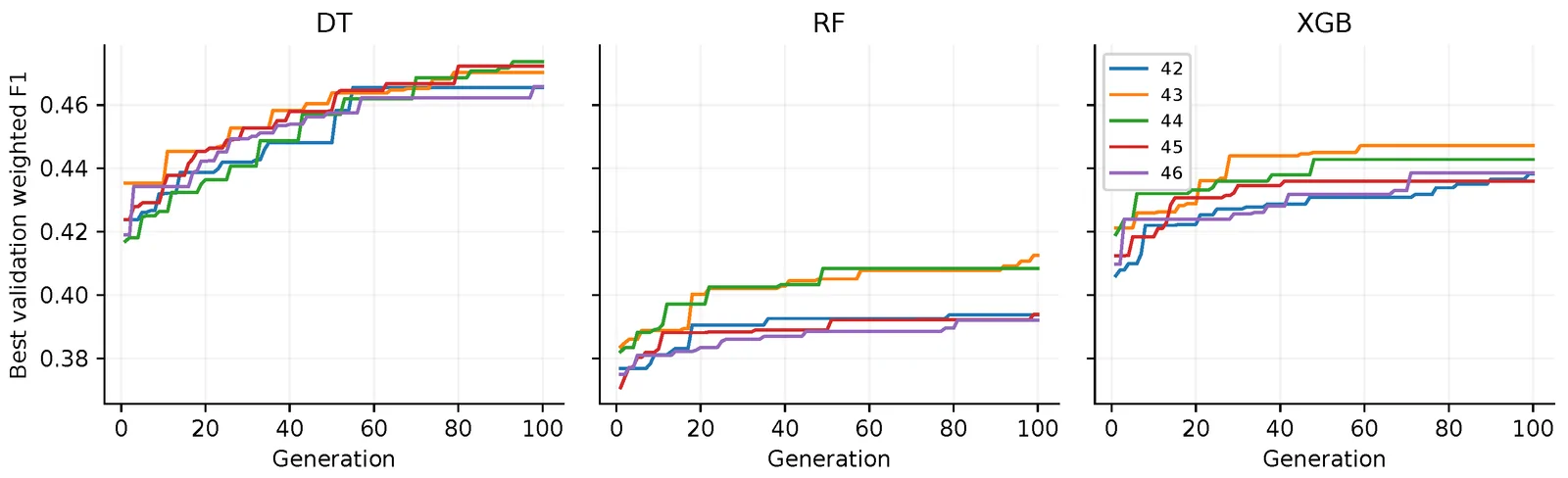
Best inner-validation weighted F1 across GA generations for the selected candidate in each outer seed.

The GA used a fixed 100-generation budget. Across the 15 selected GA runs, the final best inner-validation score was first reached between generations 41 and 99. Thus, the convergence logs do not justify a claim that 20–40 generations would always have been sufficient, nor do they establish 100 generations as an optimal stopping point. Adaptive early stopping remains a possible extension. Figure 3 shows the selected candidate’s inner-validation trajectory for each outer seed.

### 3.3. Performance of GA-Based Feature Selection

GA-based feature selection was applied to all three learners for the twelve disease targets. Table 8 and Table 9 summarize the target-wise accuracy, disease-positive precision, recall, and F1-score after GA- and PSO-based feature selection, respectively. The disease-specific results show why the aggregate accuracy values require caution. For example, only 36 dementia-positive respondents remain in the complete analytical cohort, with seven in each saved outer test set. Full Random Forest achieved high mean dementia accuracy but mean positive recall of only 0.029; the GA- and PSO-selected Random Forest variants had zero mean recall. Similar effects occur for other low-prevalence conditions. Accordingly, high accuracy for low-prevalence targets should not be interpreted as reliable disease-positive detection.

These results also show that the differences between full and selected models are generally small relative to the severe class imbalance. The feature selectors were not combined with class weighting, resampling, or threshold optimization in these experiments. The five seed-wise results are reported descriptively with mean and standard deviation. Because the repeated outer splits overlap and pooled disease/model values do not constitute independent replications, no inferential significance test is reported.

We also performed PSO-based feature selection for comparison with the GA-based method. Table 9 presents the results of applying PSO to the same multi-label classification task across the three models. Overall, PSO showed mixed results.

### 3.4. Comparison with Explainable AI Frameworks

We applied SHAP (SHapley Additive exPlanations) [47] to the fitted full, GA-selected, and PSO-selected versions of Decision Tree, Random Forest, and XGBoost for all five outer seeds. SHAP was calculated using each seed’s 906 held-out outer test records. The saved outputs include global mean absolute SHAP, disease-specific mean absolute and mean signed SHAP, and disease-specific top-30 rankings. Therefore, the selected-model explanations correspond to the actual reduced-input estimators rather than to the full-feature models alone. To clarify, SHAP analysis was not intended to identify disease-specific clinical determinants. Instead, it was used as a post-hoc analytical tool to examine whether the attribution structure of the fitted models corresponded to the feature subsets identified independently by GA and PSO, and whether this correspondence was stable across repeated data splits.

**Figure 4:**
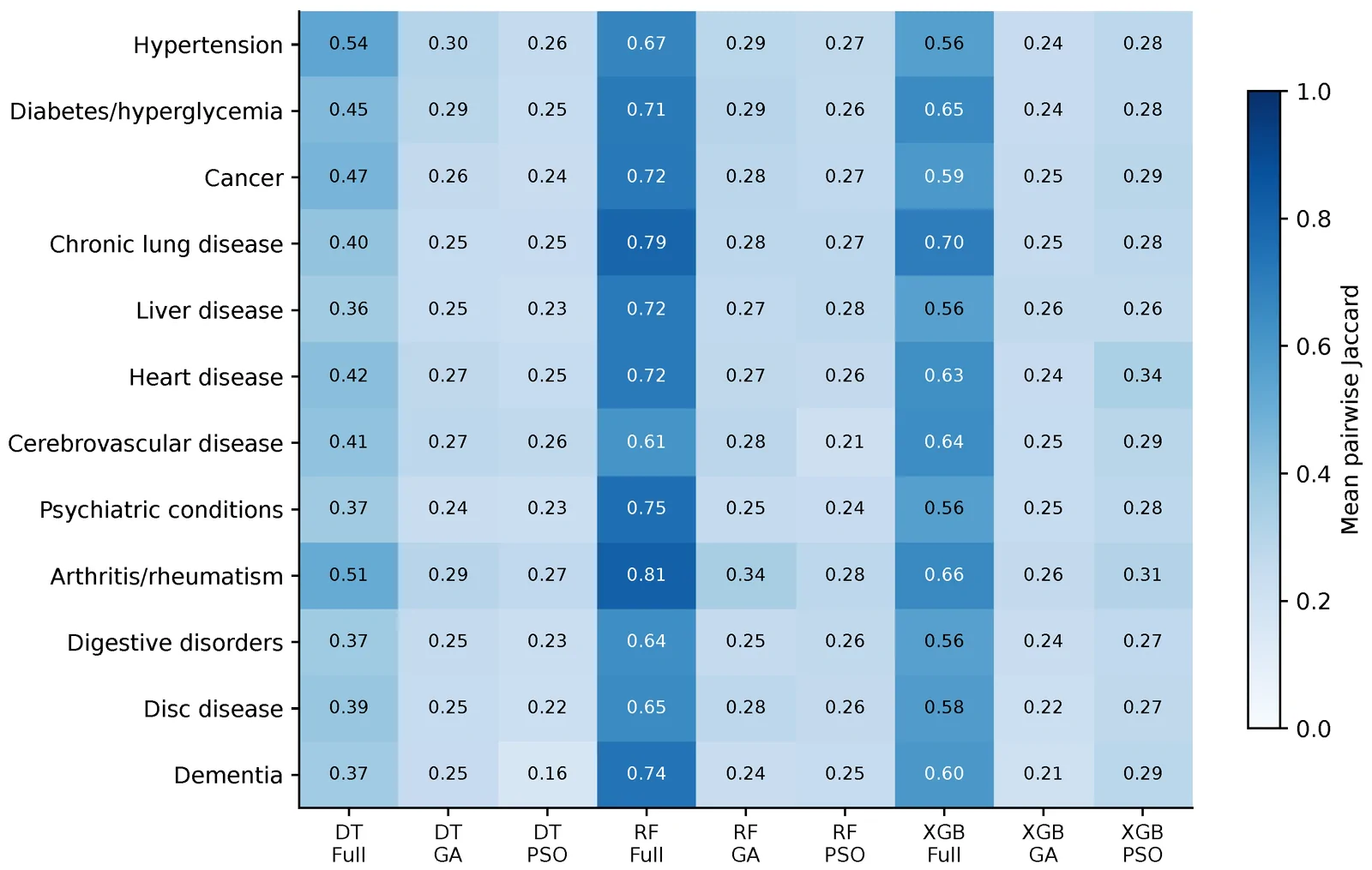
Mean pairwise Jaccard similarity of disease-specific SHAP top-30 feature rankings across seeds. Higher values indicate greater agreement in feature membership.

The stability of the top-30 feature sets was assessed by pairwise Jaccard similarity across seeds. As shown in Table 10, the mean disease-specific values were 0.422, 0.711, and 0.608 for the full Decision Tree, Random Forest, and XGBoost models, respectively, whereas the GA and PSO variants ranged from 0.237 to 0.285. The selected-model rankings were therefore less stable across the repeated splits. Figure 4 visualizes these disease-level stability values.

Table 11 compares the full-model global SHAP top-30 feature sets with the GA- and PSO-selected masks. The overlap is approximately 46.7–56.0% when the denominator is the SHAP top 30, but approximately 20.9–27.7% when the denominator is the selected mask. These values describe set membership only and should not be interpreted as explained variance or causal importance. The divergence is consistent with the distinction between attribution within a fitted model and performance-driven subset selection; related disease-prediction work has likewise combined predictive modeling with explainability for feature investigation [26].

This difference reflects the contrasting optimization goals of the two approaches. SHAP explains a model’s decision-making by estimating each feature’s marginal contribution in the context of the entire model. In contrast, GA and PSO perform a global search for feature subsets that maximize predictive performance when retraining the model. As a result, SHAP may highlight features with high explanatory power that do not necessarily improve performance when used in isolation or within a reduced feature set. Likewise, GA and PSO may prioritize features that work well in combination—even if their individual SHAP values are low.

Accordingly, SHAP and metaheuristic feature selection provide complementary views of the model. SHAP characterizes attribution within a fitted estimator, whereas feature selection addresses which combinations of features are useful for the predictive objective. Their rankings and selected sets should therefore not be treated as interchangeable.

The available archive does not retain per-observation SHAP values or complete explainer/background-data settings. Therefore, SHAP is used only for descriptive, model-specific interpretation of the saved fitted models and does not support dependence-plot analysis, patient-level interpretation, causal effects, or clinical intervention targets.

## 4. Discussion

This study aimed to evaluate the accuracy and interpretability of multi-label disease prediction through metaheuristic-based feature selection and explainable AI. In our multi-label experiments, random forest, decision tree, and XGBoost were compared under full-feature, GA-selected, and PSO-selected settings, combined with SHAP to provide model transparency.

Across the repeated outer splits, no model showed a uniformly superior performance in Composite Macro-F1 and PR-AUC. Composite Macro-F1 for the full Decision Tree, Random Forest, and XGBoost models was 0.552, 0.523, and 0.544 on average, respectively, and feature selection changed these values only slightly. The high mean label accuracy observed for several models must be interpreted cautiously because an all-negative reference already achieved high accuracy under the severe class imbalance. The decision tree is therefore treated as a simple single-tree reference rather than as a strong competitive baseline. Meanwhile, to validate performance of the selection more broadly, validation with other types of machine learning algorithms is needed, such as support vector machines or deep learning.

Applying GA-based feature selection reduced the feature set but did not consistently improve predictive performance. GA did not aggressively minimize the feature set because subset size was not included in the fitness objective, variables could remain whenever they contributed to the inner-validation score. The retained subsets should therefore be interpreted as performance-oriented feature reduction rather than as minimal or uniquely necessary feature sets. No inferential significance test is reported because the pooled disease and model-level values were not independent replications. For use cases prioritizing interpretability or cost reduction, a multi-objective GA framework might be needed to penalize excessive features explicitly.

In parallel, PSO-based feature selection produced similarly small and model-dependent changes in predictive performance. These mixed results imply that the effect of a metaheuristic feature-selection strategy depends on the learner and data split, and they do not establish the general superiority of either GA or PSO. Comparisons with simpler selectors such as mutual information, L1-based selection, recursive feature elimination, Boruta, or permutation-based selection remain necessary before broader claims are made.

To complement the predictive evaluation, we employed SHAP to explain how features influenced label predictions in the full-feature and selected models. SHAP was computed on each seed’s held-out outer test records, and the selected-model explanations therefore correspond to the actual reduced-input estimators. The selected-model top-30 rankings were less stable across seeds than the full-model rankings. SHAP focuses on attribution within a fitted model, whereas GA and PSO seek feature combinations that optimize an inner-validation objective; their overlap should therefore not be interpreted as confirmation of causal or clinical importance. This distinction is also consistent with interpretable disease-prediction work that separates predictive performance from feature-level explanation [26].

This study should be interpreted as a cross-sectional methodological evaluation rather than as a clinically validated risk model. Features and disease-status labels were measured in the same KLoSA wave, and some same-wave variables may reflect treatment, healthcare use, or consequences of an existing diagnosis. In addition, complete-case deletion was applied before the repeated train/validation/test splits, no external or temporal cohort was used, and calibration, decision-curve, subgroup fairness, missing-data sensitivity, and survey-design-aware analyses were not conducted. These limitations prevent claims of immediate diagnosis, screening, early intervention, or clinical deployment.

Furthermore, because the KLoSA dataset was collected through Computer-Assisted Personal Interviewing (CAPI) [13], the proposed framework relies on structured questionnaire data rather than laboratory or imaging variables. However, the retained features include detailed survey variables that are not routinely collected during brief clinical consultations. Any future clinical translation would therefore require a smaller clinic-available feature set, prospective and external validation, calibration, subgroup evaluation, and workflow assessment before questionnaire-driven screening or decision support could be considered.

## 5. Conclusions

In conclusion, combining ensemble models (Random Forest, XGBoost) with GA- and PSO-based feature selection and SHAP interpretation provided a framework for evaluating twelve independently predicted disease-status labels. The repeated nested holdout results show that feature selection reduced the 131-feature input but produced only small, model-dependent changes in Composite Macro-F1, and did not indicate a general performance advantage for GA or PSO. High mean label accuracy also coexisted with limited disease-positive sensitivity for several rare conditions, and SHAP rankings for selected models were less stable across seeds. Future work might refine feature parsimony via multi-objective optimization, ensemble techniques [48], and alternative feature-selection methods, and it should also examine dependency-aware multi-label models, imbalance-aware learning, and longitudinal validation. Collaborating with healthcare professionals to evaluate the clinical usability and interpretability of SHAP-based explanations can be considered only after a clinically available feature set and appropriate external, prospective, calibration, and workflow validation are established. Under those conditions, future work could also examine integration with electronic health record-linked screening dashboards [49].

## Figures and Tables

**Table 1:** KLoSA’s main investigation contents.

Categorization	Key Contents
Population	Gathering detailed information about the personal and social status of the person under investigation and their spouse
Family & Employment	Collecting information on children and grandchildren and summarizing details by employment type
Health status	Covering subjective health status, chronic disease status, treatment status, daily life difficulties, accident and injury experience, aging-related conditions, health indicators, health habits, and depression information
Income	Collecting information on earned income, pension income, Social Security income and type, other income and earnings, and gross household income over the past year
Assets	Collecting information about the residence, real estate assets, financial assets, non-financial assets, assets through inheritance/gift, liabilities, and total household assets
Subjective expectations	Gathering information on financial situation, inheritance/bequest, work hours expected, changes in standard of living, government expectations, and life satisfaction

**Table 2:** Disease-positive distribution in the analytical cohort (n=4529).

Disease	Positive	Negative	Positive (%)
Hypertension	2170	2359	47.913
Diabetes/hyperglycemia	990	3539	21.859
Cancer	354	4175	7.816
Chronic lung disease	140	4389	3.091
Liver disease	135	4394	2.981
Heart disease	445	4084	9.826
Cerebrovascular disease	251	4278	5.542
Psychiatric conditions	207	4322	4.571
Arthritis/rheumatism	1183	3346	26.121
Digestive disorders	85	4444	1.877
Disc disease	81	4448	1.788
Dementia	36	4493	0.795

**Table 3:** Parameter values for machine learning algorithms in this study.

Model	Parameter	Value
Random Forest	n_estimators	100
	max_depth	None
	min_samples_split	2
	min_samples_leaf	1
	bootstrap	True
	criterion	gini
Decision Tree	max_depth	None
	min_samples_split	2
	min_samples_leaf	1
	criterion	gini
	splitter	best
XGBoost	n_estimators	100
	max_depth	6
	learning_rate	0.1
	subsample	0.8
	colsample_bytree	0.8
	gamma	0.2

**Table 4:** GA parameters setting for the experiment.

Parameter	Value
Population size	100
Number of generations	100
mutation rate	0.005, 0.01, 0.05

**Table 5:** PSO parameters setting for the experiment.

Parameter	Value
Number of particles	100
Number of iterations	100
inertia weight	0.7
cognitive coefficient	1.5
social coefficient	1.5

**Table 6:** Comparison of prediction results for tree-based classifier algorithms applied to multi-label problem solving.

Target	Random Forest				Decision Tree				XGBoost			
	Accuracy	Precision	Recall	F1 Score	Accuracy	Precision	Recall	F1 Score	Accuracy	Precision	ecall	F1 Score
Hypertension	0.732	0.688	0.807	0.743	0.645	0.626	0.643	0.634	0.728	0.681	0.812	0.741
Diabetes or hyperglycemia	0.782	0.478	0.017	0.033	0.685	0.299	0.327	0.312	0.772	0.422	0.123	0.190
Cancer and malignancies	0.922	0.000	0.000	0.000	0.852	0.106	0.118	0.112	0.921	0.300	0.011	0.022
Chronic lung disease	0.969	0.000	0.000	0.000	0.936	0.059	0.071	0.064	0.969	0.000	0.000	0.000
Liver disease	0.970	0.000	0.000	0.000	0.933	0.026	0.037	0.030	0.970	0.000	0.000	0.000
Heart disease	0.902	0.200	0.002	0.004	0.810	0.119	0.144	0.130	0.900	0.274	0.013	0.026
Cerebrovascular disease	0.944	0.200	0.004	0.008	0.899	0.132	0.148	0.139	0.944	0.367	0.016	0.030
Psychiatric conditions	0.955	0.000	0.000	0.000	0.911	0.104	0.127	0.114	0.955	0.550	0.039	0.072
Arthritis or rheumatism	0.775	0.625	0.347	0.446	0.694	0.419	0.435	0.427	0.780	0.613	0.425	0.502
Digestive system disorders	0.981	0.000	0.000	0.000	0.962	0.104	0.129	0.114	0.981	0.000	0.000	0.000
Disc-related diagnoses	0.982	0.000	0.000	0.000	0.965	0.094	0.113	0.101	0.982	0.000	0.000	0.000
Dementia	0.992	0.200	0.029	0.050	0.988	0.199	0.257	0.224	0.993	0.633	0.143	0.224
Average accuracy over all targets	0.909±0.002				0.857±0.002				0.908±0.001			
Composite Macro F1 score for all targets	0.523±0.004				0.552±0.010				0.544±0.010			
Macro PR-AUC	0.249±0.014				0.140±0.008				0.260±0.019			

**Table 7:** Selected-feature counts and search cost across five seeds.

Variant	Features	Range	Fitness Evaluations	Search Hours
DT GA	69.4±3.1	66–74	12,323 ± 293	1.10±0.05
DT PSO	65.8±9.4	55–77	10,100 ± 0	0.88±0.02
RF GA	55.4±5.9	49–65	12,197 ± 280	5.07±0.26
RF PSO	60.0±6.2	50–65	10,100 ± 0	4.19±0.12
XGB GA	59.8±3.8	54–64	12,055 ± 615	5.21±0.34
XGB PSO	68.2±9.4	55–77	10,100 ± 0	4.38±0.07

**Table 8:** Comparison of prediction results for tree-based classifier algorithms applied to multi-label problem solving after GA-based feature selection.

Target	Random Forest				Decision Tree				XGBoost			
	Accuracy	Precision	Recall	F1 Score	Accuracy	Precision	Recall	F1 Score	Accuracy	Precision	Recall	F1 Score
Hypertension	0.727	0.679	0.817	0.742	0.657	0.642	0.646	0.644	0.726	0.676	0.822	0.742
Diabetes or hyperglycemia	0.777	0.380	0.035	0.064	0.690	0.307	0.338	0.322	0.768	0.393	0.117	0.180
Cancer and malignancies	0.922	0.200	0.003	0.006	0.846	0.109	0.132	0.119	0.921	0.336	0.028	0.052
Chronic lung disease	0.969	0.000	0.000	0.000	0.935	0.048	0.064	0.054	0.969	0.200	0.007	0.014
Liver disease	0.970	0.000	0.000	0.000	0.934	0.039	0.052	0.045	0.970	0.000	0.000	0.000
Heart disease	0.902	0.000	0.000	0.000	0.815	0.118	0.137	0.126	0.899	0.183	0.009	0.017
Cerebrovascular disease	0.945	0.333	0.012	0.023	0.898	0.117	0.128	0.122	0.944	0.433	0.020	0.038
Psychiatric conditions	0.955	0.200	0.005	0.010	0.911	0.139	0.180	0.156	0.956	0.600	0.044	0.081
Arthritis or rheumatism	0.773	0.617	0.348	0.444	0.704	0.436	0.450	0.443	0.781	0.612	0.441	0.513
Digestive system disorders	0.981	0.000	0.000	0.000	0.960	0.073	0.094	0.081	0.981	0.000	0.000	0.000
Disc-related diagnoses	0.982	0.000	0.000	0.000	0.962	0.039	0.050	0.044	0.982	0.200	0.013	0.024
Dementia	0.992	0.000	0.000	0.000	0.988	0.227	0.143	0.160	0.993	0.600	0.143	0.228
Average accuracy over all targets	0.908±0.002				0.858±0.001				0.907±0.001			
Composite Macro F1 score for all targets	0.522±0.004				0.549±0.006				0.547±0.008			
Macro PR-AUC	0.238±0.015				0.136±0.004				0.250±0.016			

**Table 9:** Comparison of prediction results for tree-based classifier algorithms applied to multi-label problem solving after PSO-based feature selection.

Target	Random Forest				Decision Tree				XGBoost			
	Accuracy	Precision	Recall	F1 Score	Accuracy	Precision	Recall	F1 Score	Accuracy	Precision	Recall	F1 Score
Hypertension	0.730	0.682	0.822	0.745	0.654	0.640	0.634	0.636	0.725	0.678	0.814	0.739
Diabetes or hyperglycemia	0.781	0.482	0.032	0.060	0.698	0.325	0.352	0.337	0.767	0.387	0.121	0.184
Cancer and malignancies	0.922	0.000	0.000	0.000	0.847	0.111	0.141	0.124	0.921	0.347	0.017	0.032
Chronic lung disease	0.969	0.000	0.000	0.000	0.933	0.069	0.086	0.076	0.969	0.000	0.000	0.000
Liver disease	0.970	0.000	0.000	0.000	0.929	0.031	0.052	0.039	0.970	0.000	0.000	0.000
Heart disease	0.902	0.000	0.000	0.000	0.820	0.147	0.171	0.157	0.898	0.130	0.009	0.017
Cerebrovascular disease	0.945	0.600	0.016	0.031	0.890	0.118	0.156	0.134	0.945	0.507	0.028	0.053
Psychiatric conditions	0.955	0.000	0.000	0.000	0.917	0.128	0.141	0.134	0.955	0.280	0.024	0.045
Arthritis or rheumatism	0.775	0.623	0.357	0.453	0.692	0.414	0.419	0.416	0.775	0.600	0.417	0.492
Digestive system disorders	0.981	0.000	0.000	0.000	0.960	0.038	0.059	0.046	0.981	0.200	0.012	0.022
Disc-related diagnoses	0.982	0.000	0.000	0.000	0.961	0.082	0.125	0.098	0.982	0.000	0.000	0.000
Dementia	0.992	0.000	0.000	0.000	0.985	0.136	0.171	0.151	0.993	0.600	0.114	0.189
Average accuracy over all targets	0.909±0.002				0.857±0.004				0.907±0.002			
Composite Macro F1 score for all targets	0.523±0.002				0.550±0.012				0.542±0.010			
Macro PR-AUC	0.237±0.014				0.137±0.007				0.249±0.020			

**Table 10:** Mean pairwise Jaccard stability of top-30 SHAP feature sets across five seeds.

Variant	Global Jaccard	Disease Mean	Disease Range
DT Full	0.765	0.422	0.360–0.535
DT GA	0.324	0.265	0.242–0.301
DT PSO	0.305	0.237	0.157–0.266
RF Full	0.930	0.711	0.610–0.815
RF GA	0.303	0.278	0.242–0.341
RF PSO	0.284	0.260	0.214–0.279
XGB Full	0.894	0.608	0.559–0.703
XGB GA	0.267	0.244	0.209–0.265
XGB PSO	0.319	0.285	0.262–0.337

**Table 11:** Full-model global SHAP top-30 overlap with GA- and PSO-selected feature masks.

Variant	Intersection Count	Of Top 30 (%)	Of Selected Mask (%)
DT-GA	16.8±2.4	56.0	24.2
DT-PSO	15.0±2.7	50.0	22.7
RF-GA	15.2±3.4	50.7	27.7
RF-PSO	16.0±3.4	53.3	26.7
XGB-GA	14.0±1.9	46.7	23.4
XGB-PSO	14.4±3.8	48.0	20.9

## Data Availability

The KLoSA data analyzed in this study are available at https://survey.keis.or.kr/klosa/klosadown/List.jsp (accessed on 15 September 2026). The experimental code is provided at https://osf.io/fsh5k/overview?view_only=c0e5504951744c948da89a4ef5ce6ba7 (accessed on 15 September 2026).

## Supplementary Materials

The following supporting information can be downloaded at https://www.mdpi.com/article/10.3390/biomimetics11100716/s1. S1: feature and target inventories and cohort audit.

## Appendix A. Feature Reduction Summary Including Missing Data

We examined all variables in the original KLoSA dataset before feature scaling and model training. Variables with more than 20% missing values were removed, and the remaining dataset was processed via listwise deletion. This method results in the removal of samples which contain any missing entries. The missing-data rules produced 4540 complete records; target validation subsequently excluded 11 records with dementia response code 3, leaving the final analytical cohort of 4529 respondents. The final analytical matrix contains 143 columns, consisting of 131 features and 12 disease target labels. Because the missingness-based eligibility rules were applied before the repeated train/validation/test splits, the full-cohort missingness pattern influenced variable and record inclusion; this is acknowledged as a limitation. Table A1 summarizes the number of features before and after preprocessing and the average missing values within each feature category.

**Table A1:** Summary of missingness and feature reduction. Total feature number is the sum of the 131 features and 12 targets.

Category	Number of Features (Before)	Number of Features (After)	Mean Missing (%)	Handling
Demographics & Basic Info	18	12	1.5	Retained (listwise deletion)
Economic & Transfer-related	12	4	60	Removed (excessive missingness)
Lifestyle & Habits	20	15	3.1	Retained (listwise deletion)
Clinical & Health Indicators	67	45	2.4	Retained (listwise deletion)
Psychological Measures	14	10	3.2	Retained (listwise deletion)
Disease History	11	11	<1.0	Retained
Total	215	143	18.7 → 0.0	Rows with missing values removed
